# An approach of total endoscopic atrial myxoma resection without robotic assistance

**DOI:** 10.3389/fcvm.2025.1686711

**Published:** 2025-12-04

**Authors:** Xingming Wang, Shuai Ma, Bingbing Ma, Hourong Sun, Biao Wang, Kai Liu, Zengshan Ma

**Affiliations:** 1 Department of Cardiovascular Surgery, Qilu Hospital of Shandong University, Jinan, Shandong, China; 2 Department of Cardiovascular Surgery, Thoracoscopy Institute of Cardiac Surgery of Shandong University, Jinan, Shandong, China; 3 Department of Rheumatology, Qilu Hospital of Shandong University, Jinan, Shandong, China

**Keywords:** thoracoscopy, myxoma, beating heart, without robotic assistance, cardiac surgery

## Abstract

**Background:**

Conventional surgery presents significant challenges for elderly patients with atrial myxoma and ascending aorta calcification. This study aims to evaluate the feasibility and safety of total thoracoscopic resection of atrial myxoma.

**Methods:**

A total of 83 patients who underwent totally thoracoscopic resection of atrial myxoma between January 2013 and April 2024 were retrospectively analyzed. Three 1.0–2.0-cm thoracic incisions were made in the right chest, and all procedures were conducted under totally thoracoscopy. Right atrial myxomas were resected via a right atrium approach, while left atrial myxomas were removed through an interatrial groove approach. For the 46 patients with ascending aortic plaque or calcification, the operations were performed on the beating heart without aortic cross-clamping.

**Results:**

The totally thoracoscopic resection of atrial myxoma was successfully performed in all patients without in-hospital mortality or a switch to the sternotomy approach. The duration of operation, CPB time, and aortic cross-clamp time in the beating-heart group were shorter than in the arrested-heart group. There were no statistically significant differences between the groups in mechanical ventilation time, duration of ICU, postoperative hospital stay, and postoperative 24-h drainage volume. The rate of tracheal extraction in the operating room was higher in the beating-heart group than that in the arrested-heart group. None of the patients required a blood transfusion or experienced serious complications.

**Conclusion:**

Total thoracoscopic resection is a feasible and safe method for addressing atrial myxomas, particularly in elderly patients with ascending aortic calcification and persistent cardiac failure.

## Introduction

Myxoma is the most common primary cardiac tumor in adults, typically arising in the left atrium and accounting for approximately 80% of all cardiac tumors (1, 2). It predominantly affects individuals aged 30–60 years, with a higher incidence in women than in men (3). The disease often progresses insidiously, leading to myxoma embolism and hemodynamic disturbances. Complete surgical resection remains the only definitive treatment for cardiac myxomas (4). Historically, median sternotomy has been the standard approach for myxoma resection (5). In recent years, alternative techniques, such as small thoracotomy with or without endoscopic support, totally endoscopic cardiac surgery without robotic assistance, and fully robotic approaches, have been reported (6–8). However, these procedures present significant challenges for elderly patients with atrial myxoma and ascending aorta calcification. In our study, we retrospectively analyzed 10 years of experience using Ma's Tri-Port Chest Thoracoscopic Cardiac Surgery Technology (MTCST) for myxoma resections performed under both cardiac arrest and beating-heart conditions.

## Patients and methods

### Patient selection

Between January 2013 and April 2024, 83 patients underwent a totally thoracoscopic resection of atrial myxoma. The inclusion criteria for the study were as follows: (1) isolated myxomas in the left or right atrium confirmed by echocardiography; (2) patient age over 18 years; and (3) preoperative evaluation of the thoracic aorta, abdominal aorta, and pelvis (via CT) confirming the absence of atherosclerotic stenosis in the femoral arteries and ascending aorta and the absence of abnormal veins. Exclusion criteria were as follows: (1) concomitant cardiac procedures (e.g., valve replacement); (2) malignant tumors; (3) severe comorbidities precluding surgery (e.g., advanced renal failure); and (4) history of chest trauma, pleurisy, or previous thoracic surgeries. Among the 83 patients, 45 were women and 38 were men. Preoperative diagnoses, including the diameter and anatomical position of the myxomas as well as any associated anomalies, were determined through transthoracic echocardiography (Table 1). All patients were in sinus rhythm preoperatively. The use of totally thoracoscopic cardiac surgery technology was approved by the ethics board and technical committee of the hospital, and informed consent was obtained from the families of patients.

**Table 1:** Clinical demographics of patients in arrested-heart and beating-heart groups.

Variables	Arrested heart (*n* = 37)	Beating heart (*n* = 46)	*P*-value
Age (years)	50.72 ± 11.52	54.33 ± 9.60	0.123
Sex (male), *n* (%)	20 (54)	27 (59)	0.671
Body weight (kg)	71.80 ± 8.95	69.17 ± 9.33	0.197
Tumor length (mm)	36.15 ± 6.50	37.56 ± 5.37	0.282
LVEF (%)	61.21 ± 3.04	60.55 ± 2.54	0.284
PASP (mmHg)	27.25 ± 4.28	28.44 ± 3.66	0.176
Stroke history, *n* (%)	2 (5.41)	4 (8.70)	0.882*

* *T* < 5, Continuity correction chi-square test.

### Anesthesia

Patients were intubated with a double-lumen endotracheal tube. A transesophageal echocardiography (TEE) probe and an arterial pressure monitoring line were established.

### Surgical technique and procedures

**Figure 1:**
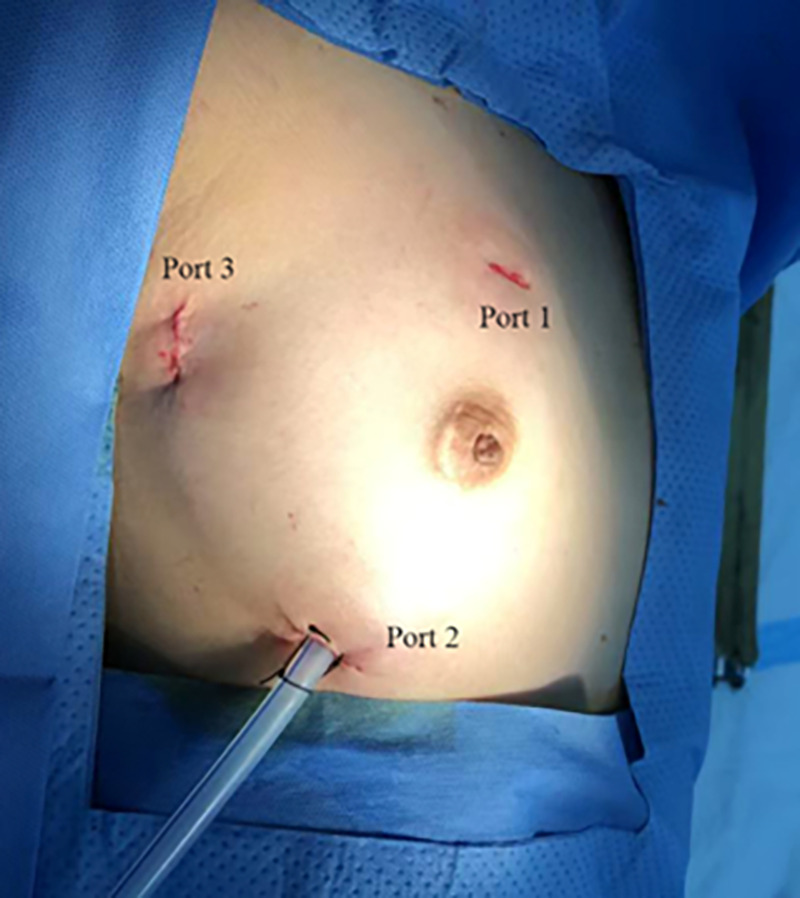
Location of the three ports in the right chest. Port 1: left-hand port; Port 2: right-hand port; Port 3: endoscopic port.

The patients were positioned supine with the right side of their body elevated by 10–15 degrees, and their right upper extremity extended along their body. External defibrillator patches were attached to the patient. Prior to the operation, incision sites for the three ports were marked. Three thoracic incisions measuring 1.0–2.0 cm were made on the right side of the chest. Port 1 (1.0–1.5 cm) was located at the right mid-clavicular line in the second or third intercostal space (ICS) for left-hand operations (used for tissue forceps and right-heart suction); Port 2 (1.0–1.5 cm) was placed lateral to the right mid-clavicular line in the fifth or sixth ICS for right-hand operations (used for forceps, needle holder, knife, and electrocautery); Port 3 (2.0 cm) was designed in the fourth ICS along the anterior axillary line for thoracoscopy (used for the endoscopic camera, left-heart suction, and external traction) (Figure 1).

The main procedures were as follows: first, Port 3 was created for thoracoscope insertion. Guided by thoracoscopy, Ports 1 and 2 were then established for instrument entry. After systemic heparinization, cardiopulmonary bypass (CPB) was established via the femoral artery and vein. Femoral arterial and venous cannulation (Medtronic, Inc.) was performed using the super-smooth guidewire method under TEE guidance. Arterial pressure was monitored, and the side ports of the bipolar femoral venous catheter were positioned in the superior and inferior vena cava, respectively. To ensure adequate venous drainage and arterial flow, vacuum-assisted venous drainage was employed with continuous monitoring of reservoir pressures (maintained at −25 to −40 mmHg). Additional internal jugular venous catheters were placed in six cases of right atrial myxoma. CPB was initiated, and the body temperature of the patients was lowered to 32 °C. Carbon dioxide was continuously insufflated into the thoracic cavity at a rate of 1 L/min to minimize the risk of embolism. The pericardium was incised longitudinally, parallel to the sternum and 1–2 cm posterior to the sternum. Traction lines were sutured to the posterior margin of the pericardium and drawn out of the chest through Port 3 to facilitate exposure of the heart. The left atrial drainage tube was correctly inserted into the right superior pulmonary vein. An additional purse-string suture was placed on the ascending aorta. For most patients, the ascending aorta was cross-clamped through Port 3, followed by antegrade perfusion of cold HTK cardioplegic solution via a 12F cannula in the aortic root. However, in patients whose ascending aorta exhibited plaque or calcification, the procedure was performed without aortic cross-clamping.

**Figure 2:**
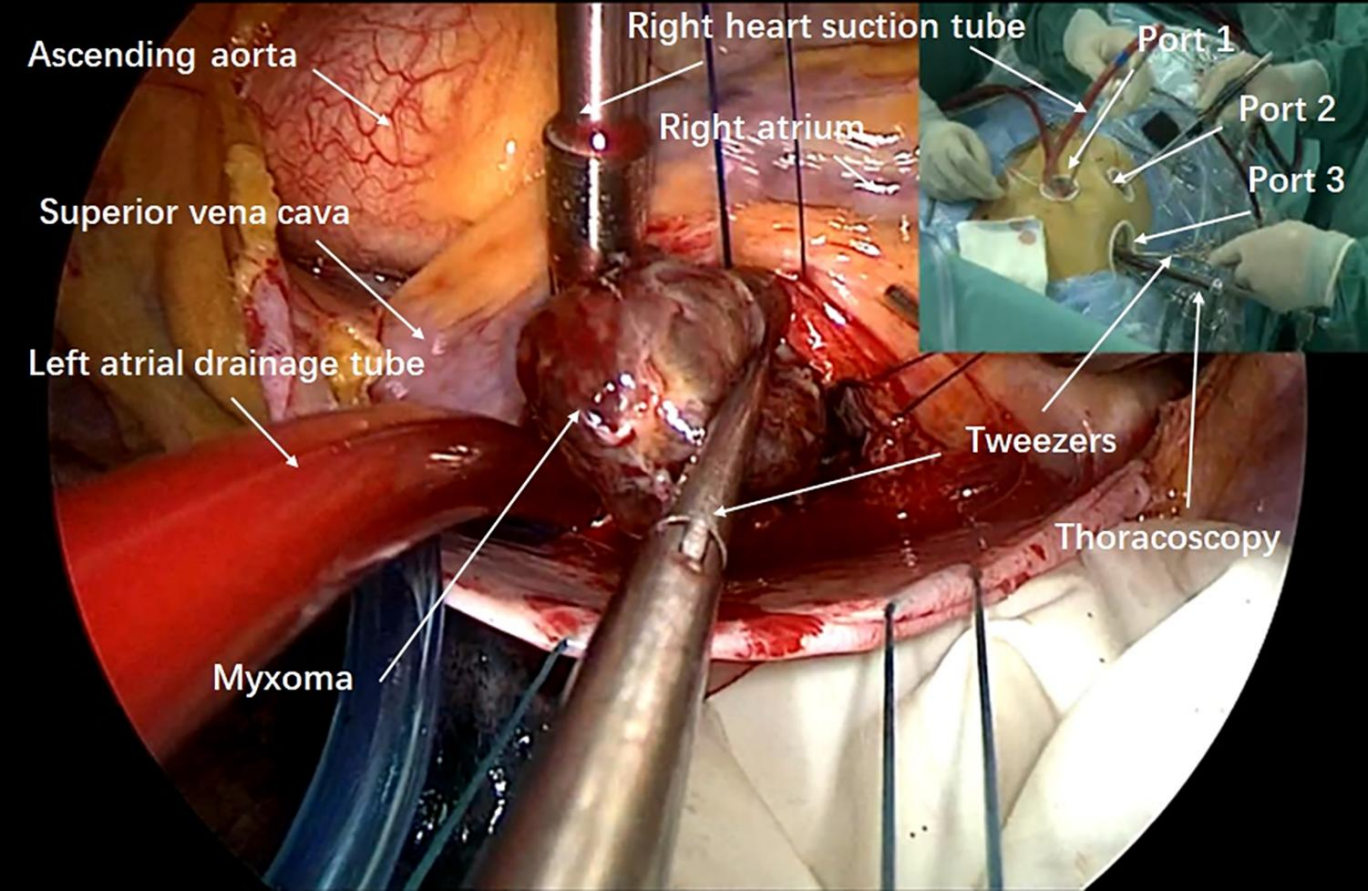
Vision of left atrial myxoma resection on screen.

An incision was made along the interatrial groove to access the left atrium. The left atriotomy was performed parallel to this groove, exposing the internal structures and the myxoma. Similar to a median sternotomy approach, lengthened vascular forceps or Alice tissue forceps were introduced through Port 1 with the left hand to grasp the tumor body or wall and stabilize it, preventing fragmentation. Meanwhile, scissors were introduced through Port 2 with the right hand to carefully dissect and cut the pedicle. Total excision was achieved by dissecting a plane through the atrial muscle at the point of attachment. All myxomas were resected completely, placed into a tumor retrieval bag, and removed via Port 1 (Figure 2). The chamber was then inspected meticulously with the endoscope. In cases where atrial septal defects were present, intraoperative repair was performed using either a Dacron patch or direct continuous suturing with 4-0 Prolene. The left atriotomy was closed with a running 4-0 Prolene suture. Before tying the knots, the left atrium was deaired by repeated inflation of the lungs with the patients in a left lateral decubitus position. TEE was used intraoperatively to confirm the absence of residual air in the left heart. The sutures were then securely tightened and knotted. The ascending aorta cross-clamp was released, allowing the heart to resume beating. Following this, the left atrial vent was removed, and a chest tube was inserted. Finally, TEE was used to verify that there were no abnormal findings in the heart.

### Perioperative management

Prior to surgery, all patients in this study received comprehensive educational and psychological counseling regarding the specifics of the surgical procedure, potential clinical outcomes, postoperative self-care, and lung exercises. Routine preoperative lung function tests were conducted for all patients. During surgery, the lungs were inflated every 20 min. Postoperatively, patients were monitored overnight in the intensive care unit (ICU), where they received low-frequency, high-volume artificial ventilation. Periodic ICU bedside chest x-ray scans were performed to rule out pulmonary complications. Once a patient's hemodynamics and spontaneous respiration stabilized, mechanical ventilation was discontinued. For patients in the beating-heart group who met all extubation criteria—(a) responding to verbal commands, (b) maintaining SpO_2_ > 95% during spontaneous respiration, (c) stable blood pressure, (d) body temperature >36.5°C, and (e) arterial blood gas parameters within normal ranges—tracheal extubation was performed at the operating table.

Intraoperative analgesia was provided using intravenous fentanyl combined with propofol for sedation. Postoperatively, a multimodal approach was employed, including patient-controlled analgesia with morphine or fentanyl and non-steroidal anti-inflammatory drugs. Pain was assessed using the Visual Analog Scale (VAS), with adjustments made to maintain VAS < 4.

### Statistical analysis

Data are presented as mean ± standard deviation (SD) for continuous variables and as number (*n*) and percentage (%) for categorical variables. Statistical analyses were conducted using SPSS 20.0 (IBM, Armonk, NY, USA).

## Results

The detailed characteristics of the patients are presented in Table 1. There were no significant differences in baseline data between the two groups. All patients underwent successful myxoma resection using MTCST. There were neither any in-hospital mortalities nor a switch to the sternotomy approach.

As presented in Table 2, the total duration of operation (107 ± 9 vs. 96 ± 6 min, *P* = 0.000), CPB time (54 ± 5 vs. 38 ± 4 min, *P* < 0.001), and aortic cross-clamp time (31 ± 5 vs. 0, *P* < 0.001) were shorter in the beating-heart group than in the arrested-heart group. There were no statistically signiﬁcant differences between the groups in mechanical ventilation time, ICU stay, postoperative hospital duration, or first 24-h drainage volume postoperatively. The rate of tracheal extubation in the operating room was higher in the beating-heart group than in the arrested-heart group. No patients required blood transfusion, and no major complications, including neurological or pulmonary complications, occurred during the postoperative hospital stay. All patients were followed up for a period ranging from 3 months to 7 years after surgery.

**Table 2:** Comparison of operational data between arrested-heart and beating-heart groups.

Variables	Arrested heart (*n* = 37)	Beating heart (*n* = 46)	*P*-value
Total operative time (min)	107 ± 9	96 ± 6	<0.001
CPB time (min)	54 ± 5	38 ± 4	<0.001
Aortic cross-clamp time (min)	31 ± 5	0	ns
Mechanical ventilation time (h)	3.21 ± 0.82	2.89 ± 0.65	0.051
Intensive care unit stay (h)	18.75 ± 3.73	18.02 ± 2.61	0.317
First 24 h drainage volume (mL)	96 ± 13	88 ± 11	0.003
Postoperative hospital stay (days)	5.6 ± 0.85	5.4 ± 0.91	0.309
Blood transfusion rate (%)	0	0	
No. of complications, *n* (%)	3 (8.1)	1 (2.2)	0.460*
Rate of extraction tube in the operating room, *n* (%)	7 (18.91)	26 (56.50)	<0.001

CPB, cardiopulmonary bypass.

* *T* < 5, Continuity correction chi-square test; ns: not significant.

## Discussion

MTCST requires only three ports in the chest wall to complete thoracoscopic cardiac surgery; therefore, it is considered a total thoracoscopy technology, a microsurgical technique, and it also falls within the category of closed cardiac surgery. MTCST primarily addresses two challenges: (1) video exposure of the surgical field: the intersection of the horizontal line of the midpoint of the sternum and the anterior axillary line of the right side was defined as the endoscopy port. A thoracoscope was inserted through this port with a mirror tilt of 30°. Excellent exposure of the heart in the video was achieved through rotational and front-to-back displacements of the thoracoscopy. (2) Special surgical instrument operation: Port 1, used for left-hand operations, is placed along the right midclavicular line, corresponding to the intercostal space of the right atrial appendage, and Port 2, used for right-hand operations, is positioned lateral to the right midclavicular line in the fifth or sixth intercostal space. In thoracoscopic heart surgery, the operation is performed using lengthened pen-type surgical instruments, which can achieve results equivalent to those of conventional direct-view surgical operations. This technique is based on the principle of intersection. In summary, total thoracoscopic heart surgery refers to cardiac procedures performed entirely through thoracoscopic techniques, visualized and guided by video imaging systems.

This study extends our previous reports that focused on other heart diseases treated with totally endoscopic cardiac surgery (9–11). We analyzed data from 83 patients who underwent atrial myxoma resection using MTCTS. All patients underwent successful operations, either on an arrested or beating heart. The patient cohort ranged in age from 28 to 82 years. Notably, all patients experienced relatively short operation and cardiopulmonary bypass (CPB) times, with no mortality or serious complications.

With increasing experience in thoracoscopic techniques, proficiency improved. First, the indications for surgery expanded from initially only adults to people over 70 years of age who could not undergo ascending aorta clamping due to plaque or calcification. These patients underwent the procedure without cross-clamping the aorta. There is very little published data currently on myxoma resection without the aid of a robotic surgical system. For instance, Deng et al. (6) reported similar thoracoscopic approaches for atrial myxomas with concurrent septal defect repair, showing similar complication rates but longer CPB times compared to our beating-heart subgroup. Robotic-assisted series, such as those by Gao et al. (7), demonstrate similar outcomes but with higher costs and longer operative times than our non-robotic technique. Beating-heart approaches in open surgery have been favorably compared for myocardial protection. In addition, tracheal extubation was performed in some patients in the operating room, and they returned to the ICU in a fully awake state. These results indicate that the thoracoscopic technique is simple and feasible.

. The procedures were carried out on the beating heart in some patients by omitting the ascending aorta cross-clamp and the myocardial protection steps involving fluid infusion. As a result, our technique required shorter CPB time and shorter operative time on beating hearts than robotic-assisted surgery (7).

Stroke is a critical concern during myxoma resection. In our study, no serious complications were observed, which may be attributed to our meticulous intraoperative protocols. Specifically, during the operation, only forceps were used to manipulate the tumor pedicle, avoiding direct touch with the myxoma body to prevent fragmentation. Before closing the atrial incision and tying the knot, carbon dioxide was fully infused into the thoracic cavity. Meanwhile, the lungs were repeatedly inflated to deair the left atrium and ventricle by placing the untightened knotted incision at the peak position. CPB atrial flow was slowly shut down, ensuring that the carbon dioxide completely dissolved in the blood before the recovery of autonomous ejection. The omission of ascending aorta cross-clamping during beating-heart myxoma resections helped avoid embolism caused by the ascending aortic plaque. Our results demonstrated minimal drainage volume (less than 100 mL on average) and a 0% transfusion rate. More than 98% of patients reported high satisfaction with cosmetic outcomes, and 76% noted that their scars were barely visible during follow-up. In addition, no patients experienced significant postoperative pain.

## Limitations

This was a retrospective observational study based on our experience with a relatively small cohort of patients at a single center rather than a randomized controlled trial, which may introduce selection bias and limit generalizability. The lack of a control group (e.g., sternotomy or robotic approaches) prevents direct comparisons. The follow-up period ranged from 3 months to 7 years, which may underreport long-term complications such as recurrence or atrial arrhythmias. In addition, the small sample size restricts subgroup analyses (e.g., by myxoma location), and potential confounders like surgeon experience were not adjusted for. Further studies with larger sample sizes, multicenter designs, and longer follow-up periods are required to establish definitive long-term outcomes.

## Conclusion

Total thoracoscopic resection is a feasible and safe method for addressing atrial myxomas, particularly in elderly patients with ascending aorta calcification and persistent cardiac failure.

## Data Availability

The raw data supporting the conclusions of this article will be made available by the authors, without undue reservation.

## References

[1] Shah IK Dearani JA Daly RC Suri RM Park SJ Joyce LD Cardiac myxomas: a 50-year experience with resection and analysis of risk factors for recurrence. Ann Thorac Surg. (2015) 100(2):495. 10.1016/j.athoracsur.2015.03.007 26070596

[2] Van Praet KM Kofler M Wilkens K Sündermann SH Meyer A Hommel M Minimally invasive extirpation of benign atrial cardiac tumors: clinical follow-up and survival outcomes. Innovations (Phila). (2023) 18(3):232–9. 10.1177/15569845231170000 37144727

[3] Schiele S Maurer SJ Pujol Salvador C Vitanova K Weirich G Meierhofer C Left atrial myxoma: imaging characteristics and clinical implications. Circ Cardiovasc Imaging. (2019) 12(3):e008820. 10.1161/CIRCIMAGING.118.008820 30813772

[4] Bernatchez J Gaudreault V Vincent G Rheaume P. Left atrial myxoma presenting as an embolic shower: case report and literature review. Ann Vasc Surg. (2018) 53:266.e13–20. 10.1016/j.avsg.2018.04.024 30012450

[5] Namana V Sarasam R Balasubramanian R Shani J. Left atrial myxoma: diagnostic and therapeutic considerations. QJM. (2016) 109(9):623–4. 10.1093/qjmed/hcw106 27402856

[6] Deng L Zhang GW Liu ZH Meng WX Liu HY. Totally thoracoscopic surgery for atrial myxomas resection and concurrent atrial septal defect repair. Eur Rev Med Pharmacol Sci. (2017) 21(3):569–75. 28239810

[7] Gao C Yang M Wang G Wang J Xiao C Wu Y Robotic-assisted excision of atrial myxoma: technical considerations and outcomes. J Thorac Cardiovasc Surg. (2010) 139(5):1282–5. 10.1016/j.jtcvs.2009.09.013 19853866

[8] Song JF Liang YP Li AG Du ZZ Zheng M Lin F Comparative study on myocardial protection during cardiac arrest versus beating heart in open heart surgery. Zhongguo Wei Zhong Bing Ji Jiu Yi Xue. (2003) 15(5):288–91. 12837189

[9] Liu K Sun H Wang B Ma H Ma B Ma Z. Feasibility and outcomes of tri-port totally thoracoscopic mitral valve replacement. Ann Cardiothorac Surg. (2021) 10(1):149–57. 10.21037/acs-2020-mv-fs-0064 33575185 PMC7867428

[10] Ma ZS Dong MF Yin QY Feng ZY Wang LX. Totally thoracoscopic repair of atrial septal defect without robotic assistance: a single-center experience. J Thorac Cardiovasc Surg. (2011) 141(6):1380–3. 10.1016/j.jtcvs.2010.10.028 21168159

[11] Wang X Sun H Ma B Liu K Ma Z. Thoracoscopic closure of atrial septal defect in perfused beating hearts. Surg Endosc. (2025) 39(1):341–8. 10.1007/s00464-024-11356-y 39548009 PMC11666695

